# Documenting the de-identification process of clinical and imaging data for AI for health imaging projects

**DOI:** 10.1186/s13244-024-01711-x

**Published:** 2024-05-31

**Authors:** Haridimos Kondylakis, Rocio Catalan, Sara Martinez Alabart, Caroline Barelle, Paschalis Bizopoulos, Maciej Bobowicz, Jonathan Bona, Dimitrios I. Fotiadis, Teresa Garcia, Ignacio Gomez, Ana Jimenez-Pastor, Giannis Karatzanis, Karim Lekadir, Magdalena Kogut-Czarkowska, Antonios Lalas, Kostas Marias, Luis Marti-Bonmati, Jose Munuera, Katerina Nikiforaki, Manon Pelissier, Fred Prior, Michael Rutherford, Laure Saint-Aubert, Zisis Sakellariou, Karine Seymour, Thomas Trouillard, Konstantinos Votis, Manolis Tsiknakis

**Affiliations:** 1 FORTH-ICS, Heraklion, Crete Greece; 2 La Fe University and Polytechnic Hospital, La Fe Health Research Institute, Valencia, Spain; 3 TIC Salut Social Foundation, Barcelona, Spain; 4 European Dynamics, Luxembourg, Luxembourg; 5 Centre for Research & Technology Hellas, Information Technologies Institute (CERTH-ITI), Central Directorate, Thermi, Thessaloniki Greece; 6 Medical University of Gdańsk, Gdańsk, Poland; 7 University of Arkansas for Medical Sciences, Little Rock, AR USA; 8 Unit of Medical Technology and Intelligent Information Systems, Department of Materials Science and Engineering, University of Ioannina, Ioannina, Greece; 9 Centre for Genomic Regulation (CRG), The Barcelona Institute of Science and Technology, Barcelona, Spain; 10 Quantitative Imaging Biomarkers in Medicine, Quibim, Valencia, Spain; 11 Artificial Intelligence in Medicine Labm Universitat de Barcelona, Barcelona, Spain; 12 Timelex BV/SRL, Brussels, Belgium; 13 Hospital Universitario y Politécnico La Fe, Grupo de Investigación Biomédica en Imagen IIS La Fe, Valencia, España; 14 MEDEXPRIM, Labège, France

**Keywords:** Data anonymization, Radiology, Radiological imaging

## Abstract

**Abstract:**

Artificial intelligence (AI) is revolutionizing the field of medical imaging, holding the potential to shift medicine from a reactive “sick-care” approach to a proactive focus on healthcare and prevention. The successful development of AI in this domain relies on access to large, comprehensive, and standardized real-world datasets that accurately represent diverse populations and diseases. However, images and data are sensitive, and as such, before using them in any way the data needs to be modified to protect the privacy of the patients. This paper explores the approaches in the domain of five EU projects working on the creation of ethically compliant and GDPR-regulated European medical imaging platforms, focused on cancer-related data. It presents the individual approaches to the de-identification of imaging data, and describes the problems and the solutions adopted in each case. Further, lessons learned are provided, enabling future projects to optimally handle the problem of data de-identification.

**Critical relevance statement:**

This paper presents key approaches from five flagship EU projects for the de-identification of imaging and clinical data offering valuable insights and guidelines in the domain.

**Key Points:**

- ΑΙ models for health imaging require access to large amounts of data.
- Access to large imaging datasets requires an appropriate de-identification process.
- This paper provides de-identification guidelines from the AI for health imaging (AI4HI) projects.

## Introduction

Artificial Intelligence (AI) is revolutionizing the realm of medical imaging, holding the potential to shift medicine from a focus on ‘sick care’ to a new era of personalized healthcare and preventive medicine. In the domain of cancer detection, treatment, and management, AI presents solutions to pressing and unmet needs, not only enhancing patient survival rates but also improving their overall quality of life. The global interest in AI applications, particularly in imaging, is soaring; driven by the availability of extensive datasets (commonly referred to as “big data”), remarkable advancements in computing power, and the emergence of innovative deep-learning algorithms.

Amidst this paradigm shift, the imaging community faces both opportunities and challenges. Apart from developing novel AI methods, there is a need to establish a shared nomenclature, devise efficient ways to store, share, and curate the imaging data [1], and also to devise effective and efficient methods for data de-identification so that it can be widely used. By collectively addressing these factors, the medical field can fully harness the transformative potential of AI in medical imaging and foster a future where healthcare becomes truly personalized and preventive [2].

In this direction, five EU projects (PRIMAGE, CHAIMELELON, ProCAncer-I, INCISIVE, and EuCanImage) are working together under the AI4HI (AI for Health Imaging) initiative [1, 3, 4], sharing experience and good practices towards the development of big data infrastructures that will enable European, ethical- and GDPR-compliant, quality-controlled, cancer-related, medical imaging, and other contextual clinical data platforms, in which both large-scale data and AI algorithms will co-exist. Although past papers out of those projects focused on considerations for AI [1], the infrastructures required for storing medical imaging data [3, 5] and the common data models selected [4], from the inception of those projects, it was clear that medical data, including images, are sensitive and fall under the scope of GDPR; as such, a specific paper dedicated to this dimension was missing. Before using the collected data in any way, the data needs to be modified to protect the privacy of the patients. To comply with legal requirements advocated by GDPR and others, all the projects had to develop an understanding of the terms of data anonymization and pseudonymization. This was a prerequisite to using the correct legal terminology and implementing the appropriate technical processes to comply with the regulation.

Anonymization is not a legally defined term; however, it can be inferred that it pertains to the process of ensuring that information is anonymous. Anonymous information is one that does not relate to an identified or identifiable natural person or to personal data rendered anonymous in such a manner that the data subject is not or no longer identifiable. To determine whether a natural person is identifiable, an account should be taken of all the means reasonably likely to be used, such as singling out, either by the controller or by another person to identify the natural person directly or indirectly (recital 26 GDPR).

Pseudonymization is the processing of personal data in such a manner that the personal data can no longer be attributed to a specific data subject without the use of additional information, provided that such additional information is kept separately and is subject to technical and organizational measures to ensure that the personal data are not attributed to an identified or identifiable natural person (Article 4(5) GDPR).

In addition to the terms above, a related concept is “data de-identification”, which is not legally defined by GDPR. In the literature, it is used as a term encompassing both anonymization and pseudonymization. In the recently published ISO/IEC 27559:2022 standard regarding data de-identification, it is said that “de-identification is one potential means for facilitating the use of personally identifiable information in a way that does not identify or otherwise compromise the privacy of an individual or a group of individuals”. Also in this article, we will use the term “de-identification” in this meaning.

In this paper, we explore the approach for de-identification of the five AI4HI projects comparing and contrasting their approaches and providing useful guidelines for future projects in the domain. Relevant surveys on image de-identification already exist [6–14], however, in this paper, we present de-identification of both imaging and related clinical data through the views of five EU projects, offering key insights into the approach of these five flagship projects in the domain.

The remainder of this paper is structured as follows: in the “Methods” section we report the approaches followed by the five projects, whereas in the “Results” section, we contrast the approaches of these projects offering valuable insights. The “Discussion” section concludes this paper.

## Methods

In order to document the de-identification process of clinical and imaging data for the AI4HI projects we carefully examined the de-identification process followed in each one of the five projects, documenting the process followed and reporting key steps. Details on the de-identification process of all projects are reported in Appendix 1, where we report for each project the approach specifically for de-identifying imaging and related clinical data, documenting also challenges and solutions by the individual projects:

### PRIMAGE

This project proposes an open cloud-based platform to support decision-making in the clinical management of two pediatric cancers, neuroblastoma, the most frequent solid cancer of early childhood, and diffuse intrinsic pontine glioma the leading cause of brain tumor-related death in children.

### CHAIMELEON

The CHAIMELEON project aims to develop a structured repository of health images and related clinical and molecular data on the most prevalent cancers in Europe: lung, breast, prostate, and colorectal. It aspires to deliver and Establish an EU-wide interoperable repository with quality-checked imaging data as a resource for developing and testing AI tools for cancer management.

### ProCAncer-I

ProCAncer-I’s vision is to deliver a prostate cancer AI platform featuring a unique collection of mpMRI images worldwide, in terms of data quantity, quality, and diversity. It focuses on delivering novel AI clinical tools based on a three-stage ensemble modeling process for advancing the characterization of lesions, assessment of the metastatic potential, and early detection of disease recurrence.

### INCISIVE

The INCISIVE project aims to develop a toolbox for enhancing the accuracy, specificity, and sensitivity of existing cancer imaging methods. The idea is to generate a pan-European repository of medical images that can be used for ML-based training for various types of cancer. The project’s deliverables will assist in the accurate prediction of tumor spread, evolution, and relapse, in addition to helping stratify patients.

### EuCanImage

The goal of the EU-funded EuCanImage project is to build a secure, large-scale European cancer imaging platform with capabilities that will advance the application of AI in oncology, enabling the investigation of unmet clinical needs, such as the detection of small liver lesions and metastases of colorectal cancer or the evaluation of the molecular subtypes of breast tumors.

## Results

This section collects, organizes, and compares part of this information so that the reader can have a generic idea of how each project manages the data de-identification. First, we contrast the approaches for the de-identification for the various stages in Table 1 showing that depending on the approach various policies were applied to de-identify the patient IDs usually followed by a specific naming convention or hashing. Regarding images, the de-identification process focused on retaining the required tags only and removing ones with personal information. Finally, for the clinical data, most of the projects relied on eCRFs where clinical data that was recorded did not contain personal information. In the case of dates, they were also shifted to keep longitudinal information or transformed so as not to reveal the actual date of the study.

**Table 1:** De-identification approaches of the various AI4HI projects

Project	Identifiers	Images	Clinical data	Approach
CHAIMELEON	1) Patient identifiers are randomly generated and linked to their original patient identifier. 2) New patient identifiers are generated and no table of correspondence is kept.	CTP anonymizer offers the customization of the de-identification of each of the DICOM tags in an image based on attribute confidentiality profiles of the DICOM Standard PS3 Part 15.	1) In the pseudonymization phase dates are kept. 2) Then all dates are shifted to keep longitudinal information.	Only fully anonymized data at a central repository. Tools: Medexprim Suite and CTP anonymizer.
EuCanImage	Pseudonymization by encrypting the patient’s medical record ID using CMRAD producing a unique EuCanImage-ID.	Removes specific DICOM tags containing personal information. DICOM tags that might be clinically relevant such as the patient’s age or weight are kept and modified according to the study protocol.	Collected data using REDcap and do not contain direct identifiers. Indirect identifiers were modified (e.g., date of diagnosis replaced by age at diagnosis, etc.).	Pseudonymized data in a set of repositories. Tools: CMRAD, REDcap, Euro-BioImaging XNAT, and EGA.
INCISIVE	A naming convention was proposed for the patient ID.	Removes specific DICOM tags containing personal information using a CTP anonymizer.	Keep the original offset between consecutive examinations of the patient the same after the de-identification process.	Hybrid repository (federated nodes and central node) storing pseudonymized data (during the term of the project), data to be anonymized for post-project data re-use. Tools: CTP anonymizer.
ProCAncer-I	The case’s final identity is defined by the parameter PCa followed by a hash string generated from the site ID and the original patient ID and is attributed to each case during the second anonymization stage.	1) First step anonymization (blacklisting) performed at local sites. 2) The second step (whitelisting) ensured that only specific DICOM tags were preserved.	Collected data through an eCRF form that does not contain direct identifiers. Indirect identifiers were modified (e.g., date of diagnosis replaced by age at diagnosis, etc.).	Fully anonymized data is stored in a central repository. Tools: CTP Anonymizer.
PRIMAGE	During the data ingestion process, the PRIMAGE platform generates a new code, following a specific structure for local IDs. For cloud IDs the EUPID is used for pseudonymization.	Sensitive information as stated in the DICOM standards PS3.15 is removed or emptied from the uploaded files. In addition, the PRIMAGE platform includes a tool to remove any sensitive burned data within the image.	Clinical data is ingested through an eCRF or automatically through APIs. Sensitive fields, such as the birthdate, are transformed. Free text fields are avoided.	Pseudonymized data is stored in a central repository. Tools: EUPID.

*EGA* European genome archive, *EUPID* European unified patient identity

**Fig. 1:**
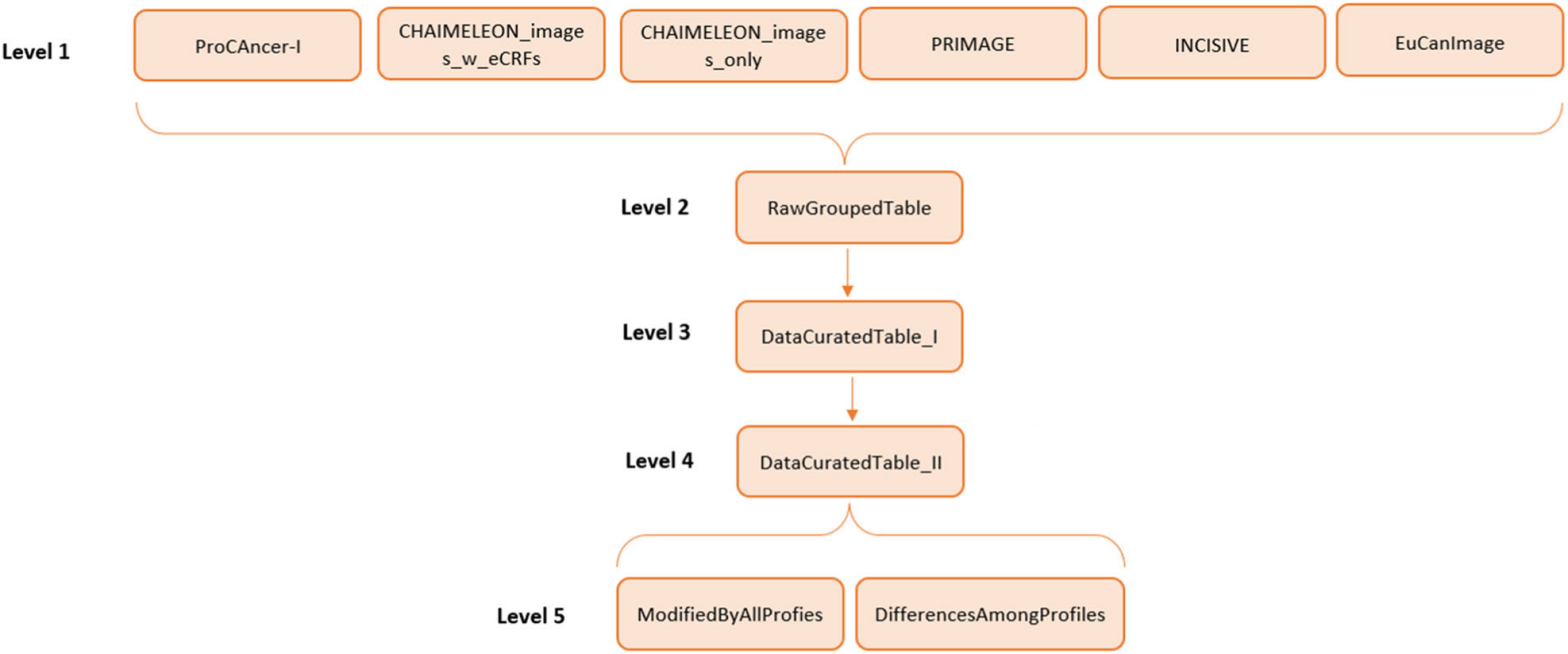
Imaging data used in the AI4HI projects

More specifically regarding imaging data, we present an additional comparison that has been made over imaging data as DICOM images are used in all the projects. DICOM metadata are very homogeneous compared to clinical data, as they are structured in tags, which has facilitated their comparison. Nonetheless, it was meticulous work to curate and align all the information as each project described it in a different way. Figure 1 presents a diagram of the process: from the information each project provided (level 1) to the final tables which contain the comparison commented before (level 5). As it was described in the “Methods” section, imaging data in the CHAIMELEON project are de-identified in two different ways depending on whether these images are accompanied by their eCRFs (structured clinical data) or not, so both of them have been treated separately from level 1.

Hereafter, level 5 of Fig. 1 will be discussed. At this level, the actions performed over the different DICOM tags have been reduced to Modify or Keep. Modify action encompasses a lot of techniques such as hashing, date modification, or pseudonym substitutions. Keep action has been attributed to those tags whose information has not been altered.

The ModifiedByAllProfiles table (Table S1 at Appendix 2) contains the 47 DICOM tags that have been modified by all these de-identification approaches. These six de-identification methods have detected them as potentially containing personally identifiable information.

The DifferencesAmongProfiles table contains the 4568 DICOM tags that have not been treated equally by these six methods, in other words, at least one of the projects has taken a different decision over them. Table 2 summarizes some figures in the DifferencesAmongProfiles table, and some conclusions can be drawn. ProCAncer-I approach is the most conservative one, as it modifies 1333 tags that the rest of the projects keep unaltered. Moreover, CHAIMELEON, PRIMAGE, INCISIVE, and EuCanImage projects modify approximately 140–400 DICOM tags, apart from the 47 tags in the ModifiedByAllProfiles table (Table S1 in Appendix 2).

**Table 2:** Modifications by the various AI4HI projects in the DifferencesAmongProfiles table

	ProCAncer-I	CHAIMELEON _images_w_eCRFs	CHAIMELEON _images_only	PRIMAGE	INCISIVE	EuCanImage
N° tags modified by this profile	4526	139	176	143	325	403
N° tags only modified by this profile	1333	0	0	0	0	0

Associations such as NEMA, which maintains the DICOM standard, or Radiological Society of North America, through its CTP Anonymizer app, propose different methods to de-identify DICOM images. These de-identification profiles can be used as a starting point to shape the de-identification method that better suits a specific project. On the one hand, there are some tags that can be used to directly identify a patient. These tags must be processed in all cases through truncating, rounding, or removal, among other de-identification techniques. On the other hand, there are tags that, not by themselves but in combination with others, can be used to determine the identity of a subject. For instance, a person could be identified by combining the information of the postal code and the age. In addition, the AccessionNumber tag may contain the hospital identifier, which, in combination with a specific pathology, could identify a patient. Each project has defined a different de-identification approach in terms of the actions taken on the tags. Despite the heterogeneity of objectives and methods among the projects, each one has its own technical committee and Data Protection Officer who have validated that the remaining tags cannot be used to reidentify the patients, and who continuously review and give support about these aspects.

### Legal aspects

As often discussed in the context of using health data and genetic data for scientific research purposes, there is a lack of universally agreed criteria on the qualification of de-identified data as either anonymized information (i.e., non-personal data) or as pseudonymized data (i.e., personal data). In addition to the technical challenges outlined in this article, there is an ongoing academic discussion focused on the interpretation of the key element of the definition of personal data (i.e., the phrase ‘means reasonably likely to be used to identify the natural person’), which is fueled by conflicting views of EU Data Protection Authorities and the courts on the matter. This lack of agreement over the concepts of anonymous and pseudonymous data is illustrated by a recent judgment of April 23, 2023, of the General Court (case T 557/20), in which the court assessed the status of data from the position of the recipient of the data and the information available to this recipient. Also, the European Data Protection Board (an administrative body that under GDPR provides guidance on the interpretation of GDPR) did not issue guidelines on anonymization under GDPR and the prior guidelines issued under Data Protection Directive 95/46/EU have been criticized as being overly restrictive.

Against this backdrop, the advancements in technology and the increase in available computational resources have led to new concerns about privacy risks in sharing any medical data with the broader community. If the de-identified medical data, which is initially considered as ‘anonymous’, is shared without proper controls and restrictions, this may lead to the risk of its re-identification by a motivated attacker. With this in mind, the legal framework for sharing medical data has to be designed to protect all the contributed data, even if considered as ‘anonymous’ during the data submission. The process needs to consider the sensitivity of the shared medical information and the potential of linking the data to the patient (for example, with the use of future technologies). These challenges were embraced by each project and translated into actionable agreements, processes, and policies.

We will present two indicative scenarios from two of the projects on how these issues are tackled.

In EuCanImage, the team worked with each clinical site (the data controller) to establish necessary Data Transfer Agreements and other legal agreements in accordance with the “Data processing and sharing based on data provider’s decisions and instructions,” scenario for the centralized image analysis model. Data Processing and Sub Data Processing agreements were put in place between the data controllers and CMRAD, and between CMRAD and Euro-BioImaging. Moreover, each clinical site has signed a Data Processing Agreement with Euro-BioImaging and the European Genome Archive for the purpose of storage of research data that regulates instructions for processing and sharing of the data they control. To enable the use of the research data all clinical sites also signed the DPAs with each AI developer individually based on the participation in the project use cases, intended uses of the data, and bilateral agreements for data processing. Discussions between legal WP1 and the clinical working group allowed the identification of the two major opposing requirements related to data de-identification and GDPR compliance. As the project is working with the retrospective data collected during the standard of care procedures, with finalized events, it would be most convenient to fully anonymize all the research data and stay outside of the GDPR regulation. Nevertheless, health information is highly specific and sensitive. Some might argue that the de-identified DICOM files still contain a lot of personal or otherwise sensitive information that, combined with supplementary clinical information and the use of sophisticated bioinformatic tools, might allow re-identification. In such a case the ‘Privacy rights for data subjects’ section of the GDPR specifies requirements for data controllers and processors. One of them is Art. 17 GDPR’s ‘Right to erasure (‘right to be forgotten’)’. Due to the multi-step procedure of project-ID generation with multiple players having encrypted information from only a single step, it is a one-way procedure allowing clinical sites to erasure individual case data when such a request is processed. Nevertheless, this right cannot be executed with respect to data already used to train algorithms.

In INCISIVE, on the other hand, the clinical partners carefully minimized the contributed datasets, removed the directly identifying information, and defined indirect identities, with the use of the described “de-identification” tools. It was concluded that the most appropriate term for the tools used in INCISIVE is “de-identification tools”. These tools assist the controller in removing or altering certain identifiers in the medical data set. However, the controller needs to assess whether the data is anonymous or pseudonymous based also on all of the circumstances relating to the possibility of re-identifying the patient (those circumstances may also relate to having access to other sources of information about the patient, for example). It was concluded that the term “anonymization tool” may be perceived as falsely assuring the controller that the data, once passed through the tool, will always become anonymous. Hence, despite the rigorous process described above, for the course of the project, most of the datasets were cautiously considered as ‘pseudonymized’ rather than ‘fully anonymized’. This also allowed a gateway for the partners to add new data to the patient record or correct it, if needed. The project applied rules provided in the GDPR for processing personal data during the project course. Accordingly, the partners entered into joint controller agreements and processing agreements, which uphold the GDPR standard. In particular, for the sharing of data between a defined group of beneficiaries (i.e., INCISIVE Data Providers and Data Users), bound by a clear purpose of implementation of the action and using defined means of processing, the joint controllership arrangement seemed most appropriate. In the experience of INCISIVE, this agreement model proved effective in providing legal safeguards and yet flexible enough to allow necessary sharing of data between the partners. INCISIVE has also developed a legal framework for the future sharing of anonymized health data, which will be relevant to the INCISIVE sustainability efforts and re-use of data from the project in the future.

## Discussion

There is limited technical literature concerning the de-deidentification of medical information. Most of the bibliography simply addresses the need for de-identification both in medical and imaging data. However, only a few of them specifically consider DICOM tags with personal health information [15]. In the following list, there are 50 attributes that were selected in that paper because of their potential ability to cause a security breach by giving data to a third party, either independently or in combination with other attributes. The comparison of ten de-identification tools in the performance over these tags has led to some conclusions: In the default configuration of the different tools, only two tools provided a high success rate: 100% in the case of DICOM Library and 98% in CTP. The rest of the tools achieved a good rate after the customization of the application.

**Table:** 

0008,0020 StudyDate	0008,1060 NameOfPhysicianReadingStudy
0008,0021 SeriesDate	0008,1062 PhysicianReadingStudyID Sequence
0008,0022 AcquisitionDate	0008,1070 OperatorsName
0008,0023 ContentDate	0010,0010 PatientsName
0008,0024 OverlayDate	0010,0020 PatientID
0008,0025 CurveDate	0010,0021 IssuerOfPatientID
0008,002A AcquisitionDateTime	0010,0030 PatientsBirthDate
0008,0030 StudyTime	0010,0032 PatientsBirthTime
0008,0031 SeriesTime	0010,0040 PatientsSex
0008,0032 AcquisitionTime	0010,1000 OtherPatientIDs
0008,0033 ContentTime	0010,1001 OtherPatientNames
0008,0034 OverlayTime	0010,1005 PatientsBirthName
0008,0035 CurveTime	0010,1010 PatientsAge
0008,0050 AccessionNumber	0010,1040 PatientsAddress
0008,0080 InstitutionName	0010,1060 PatientsMothersBirthName
0008,0081 InstitutionAddress	0010,2150 CountryOfRresidence
0008,0090 ReferringPhysicians Name	0010,2152 RegionOfResidence
0008,0092 ReferringPhysiciansAddress	0010,2154 PatientsTelephoneNumbers
0008,0094 ReferringPhysiciansTelephoneNumber	0020,0010 StudyID
0008,0096 ReferringPhysicianIDSequence	0038,0300 CurrentPatientLocation
0008,1040 InstitutionalDepartmentName	0038,0400 PatientsInstitutionResidence
0008,1048 PhysicianOfRecord	0040,A120 DateTime
0008,1049 PhysicianOfRecordIDSequence	0040,A121 Date
0008,1050 PerformingPhysiciansName	0040,A122 Time
0008,1052 PerformingPhysicianIDSequence	0040,A123 PersonName

The above-mentioned article does not evaluate the suitability of the 50 DICOM tags chosen in relation to the possibility of containing personal health information, but rather the performance of the tools when acting on these tags. These DICOM tags were chosen since they contained data that could be used to reconstruct a patient’s identity individually or in combination with other tags although it has not been assessed how these labels could be used to re-identify patients. The lack of a technical bibliography on how to de-identify medical data and the different needs of the projects encourages us to keep on working in search of the best methods to properly and securely de-identify clinical and imaging data [16–19].

## Conclusions

Developing AI-based applications in healthcare imaging relies on the availability of well-curated and annotated large datasets. This highlights the need to store and share data in an efficient manner and follow the ethical and legal regulations that will allow the reusability of the data.

In this paper, we have explored and analyzed the data de-identification approach for the AI4HI projects (Primage, Chaimeleon, ProCancer-I, Incisive, EuCanImage). Under the scope of these projects, there has been deep research on the best practices for data anonymization and pseudonymization. Overall, each project has defined a different de-identification approach in terms of the actions taken on the tags. Despite the heterogeneity of objectives and methods among the projects, each one has its own technical committee and Data Protection Officer who have validated that the remaining tags cannot be used to reidentify the patients, and who continuously review and give support about these aspects

In pursuit of enhanced collaboration, standardization, interoperability, and trust in data sharing, a new project has been raised from the AI4HI initiative: EUCAIM (European Federation for Cancer Imaging). This collective effort aims to amplify the impact of the five AI4HI projects and drive AI in cancer imaging to new heights. The EUCAIM project will link resources and databases in a federated manner facilitating the reusability of the data from the projects as well as empowering the inclusion of new additional datasets. It provides an extraordinary opportunity for the imaging community to have the largest cancer-related imaging repository in the EU. On the other hand, it also faces the important challenge of achieving this while maintaining a secure and privacy-preserving infrastructure.

### Supplementary information

ELECTRONIC SUPPLEMENTARY MATERIAL

## Data Availability

Not applicable.

## Abbreviations

AI: Artificial intelligence
AI4HI: AI for health imaging
CMRAD: Collective minds radiology
CTP: Clinical trial processor
DICOM: Digital imaging and communications in medicine
eCRF: electronic case report form
EU: European union
GDPR: General data protection regulation
