# Supplementary material for: Documenting the de-identification process of clinical and imaging data for AI for health imaging projects
Source: Insights Imaging. 2024 May 31;15:130. doi: 10.1186/s13244-024-01711-x (PMC11139818; doi:10.1186/s13244-024-01711-x)
Supplement: Supplementary file 1 — ELECTRONIC SUPPLEMENTARY MATERIAL [file 13244_2024_1711_MOESM1_ESM.pdf]

Documenting the De-identification Process of clinical and imaging data for AI for Health Imaging projects

ELECTRONIC SUPPLEMENTARY MATERIAL

Appendix 1

CHAIMELEON

De-identification process

CHAIMELEON stores only anonymized data (including clinical and imaging data) on the central repository. The main reason for this decision is to facilitate the acquisition of ethics committee approval at clinical sites. This is because even though the condition for this approval differs among institutions and regulations, providing anonymized rather than pseudonymized data to the project has proven to be a major key factor for Ethical Committee approval in most cases. As a consequence, there is not any table of correspondence kept between the original "patient ID" and the CHAIMELEON "subject ID", and only complete and fully curated cases are sent to the central repository.

Identifier anonymization. The anonymization of DICOM images in CHAIMELEON is done in two steps, as it will be described later. In each step, a new identifier is generated. Firstly, patient identifiers are randomly generated and linked to their original patient identifier in a table of correspondence. Later on, in the second step, new patient identifiers are generated and no table of correspondence is kept. This second action is crucial to consider the data anonymized before sending it to the central repository.

Image anonymization. The de-identification process for DICOM images is a two-step process as illustrated in Figure 2. As it was mentioned earlier, only anonymized information leaves the healthcare environment (clinical site) to the research environment (central repository). In order to achieve this, these two steps are carried out in the healthcare environment via the Medexprim Suite [**Error! Reference source not found.**] tool:

Step 1. Pseudonymization: DICOM images are pseudonymized in this first step in two different ways depending on whether these images are accompanied by their eCRFs (structured clinical data) or not. In both cases, CTP Anonymizer is the de-identification tool used. CTP Anonymizer offers the customization of the de-identification of each of the DICOM tags in an image. For each of these cases, one script has been developed. Both of them are based on the chapter "E. Attribute Confidentiality Profiles" of the DICOM Standard PS3 Part 15 and the following options are applied:

- In the images accompanied by their eCRFs script:
  - Retain Longitudinal Full Dates
  - Retain Safe Private

    - Clean Descriptors
- In the images only script:
    - Retain Safe Private
    - Clean Descriptors

In these processes, all direct identifiers are either removed or replaced by a randomly generated pseudonym. A table of correspondence is kept within the hospital and only accessible by an authorized user. Clinical data are associated with images using the same pseudonym. For images accompanied by their eCRF, all dates, including exam dates and dates of birth, are kept at this stage. Keeping original dates at this stage proved to be necessary whenever discrepancies were found during the curation process, to allow back-and-forth discussions between people in charge of the data curation (pseudonymized data) and the ones in charge of the data collection (entitled to view identifying data). For images-only, the purpose of the collection was to get imaging exams for the development of image curation and quality control tools. Hence the longitudinally between exams was not relevant, and all dates were changed.

Step 2. Anonymization: Once the data curators have checked inclusion criteria appropriateness, data consistency, and completeness (at least 12 months of follow-up exams after treatment), a new patient identifier is generated (no table of correspondence kept), and all dates are shifted to keep longitudinal information. This is done via Medexprim Suite functionalities. Only then, data is sent to the central repository.

Challenges and Solutions

The workflow that the clinical data and the images follow before reaching the central repository is not unique. Figure 3 represents two different use cases. The upper one (a) is followed by most of Chaimeleon's partners, while (b) is implemented by French partners. The main difference between them is the (non-)existence of an intermediation platform where the pseudonymization is performed. In any case, clinical and imaging data in the central repository are anonymized.

Following the recommendations from the DICOM Standard PS3 Part 15, by default, all private tags are removed from the images. A list of private tags of interest for the project has been identified by partners, which is whitelisted to be kept according to the confidentiality profile Retain Safe Private. Two additional private tags have been added to the whitelist, and are filled during the de-identification process to keep track of the origin of the images and the cancer they are related to (from the 5 cancers of the project).

EuCanImage

EuCanImage is built over existing services and repositories following a centralized model. The central infrastructure involves three key data processors, Collective Minds Radiology (CMRAD), Euro-BioImaging XNAT, and the European Genome Archive (EGA). CMRAD receives, pseudonymizes, and makes medical images accessible for image analysis, annotation, segmentation, and review. Euro-BioImaging XNAT and EGA act as core repositories to store imaging or clinical/phenotypic data, respectively. All these services need to be adequately integrated, linked, and share the same pseudo-anonymized patient IDs to correlate the different data types.

De-identification process

Identifier pseudonymization. The pseudonymization strategy followed in EuCanImage to preserve patient privacy involves encrypting the patient's medical record ID. Pseudonymization of the data is the essential first step for subsequent data sharing, and the same protocol is followed by all clinical sites for the different use cases within EuCanImage. This pseudonymization stage is performed in Collective Minds Radiology (CMRAD), a cloud-based GDPR-compliant platform, where collaborative image annotation and labeling are performed. The standard hash algorithm adopted by the platform is SHA512/256, the truncated version of SHA512 to an output of 256 bits, which at the time of implementation was the strongest algorithm commonly available fitting the DICOM standard. To generate the hash key the input information is a secret key (unique for each hospital) concatenated together with the personal patient's ID (hospital ID or any other identifier selected by the provider). At the next step, the patient's hashed ID is verified by the clinical site with a repeat procedure of hash generation that needs to match the stored hashed ID. The final patient's hashed ID is a 64 alphanumeric characters code. This code is assigned a unique EuCanImage-ID that is then commonly used by the repositories of the EuCanImage platform responsible for the final archiving of imaging data (Eurobioimaging) or clinical data (European Genome-Phenome Archive).

Image anonymization. The Digital Imaging and Communications in Medicine (DICOM®) Standard (ISO 12052:2017) defines profiles that detail what data elements contained in a DICOM information object (e.g., image, structured report, segmentation object) need to be modified and in what manner to achieve specified levels of deidentification and pseudonymization. After a rigorous comparison of DICOM deidentification tools, EuCanImage selected the tools provided by CMRAD as these were found to be DICOM and GDPR compliant and acceptable to the clinical sites. These tools were modified for site-specific data minimization according to local regulatory requirements. That way, the pseudonymization procedure removes specific DICOM tags containing any personal information, such as the name of the patient, date of birth, or contact details. At the same time, the DICOM tags that might be clinically relevant such as the patient's age or weight are kept and modified according to the study protocol.

Clinical data anonymization. Clinical data is collected using an electronic case report form (eCRF): REDcap, a secure (GDPR-compliant) software for building and managing surveys and databases. The data collected do not contain direct identifiers, only the EuCanImage ID. Additionally, to decrease the risk of identifiability we modified indirect identifiers and avoided their use when not essential for the scientific purpose. Examples are replacing the date of diagnosis by age at diagnosis, substituting starting and ending dates of procedures by periods of time, or by the use of arbitrary dates.

At one clinical site, we are piloting the use of a novel clinical data collection tool that ingests data extracted from the EMR system. Patient data are anonymized on-site in the process of extracting them to JSON files, which are then loaded into the clinical data collection tool. The tool extracts key elements, allowing the user to add or modify as needed, and exports the anonymized data for upload into the REDcap repository.

Challenges and Solutions

Burned-in information in secondary captures: CMRAD platform automatically curates uploaded imaging series allowing the detection and erasure of single images with burnt-in information from the series.

Handling of Private DICOM tags: Private DICOM tags are removed by the CMRAD tool during anonymization. During the verification of the uploaded MR series from one vendor, it occurred that private tags contained spatial information. A solution to the problem is currently being discussed.

INCISIVE

One of the main goals of the INCISIVE project is to create a hybrid repository (both central and federated) consisting of clinical data and DICOM images. Clinical data and DICOM images would be used for AI algorithms implementation (development, training & validation) and as input for various AI services in the context of cancer management and follow-up. The INCISIVE platform also includes tools to help the data providers contribute their data in a GDPR-compliant manner, in particular, to assist them with data minimization.

De-identification process

The first step towards de-identification was the identification of the type of data that needed to be de-identified. After several discussions between the data providers and other relevant partners of the project, the project concluded that the repository would only consist of DICOM data. As such the DICOM protocol and possible de-identification techniques and protocols were examined, namely the NEMA and TCIA protocols, and available tools. Also, other tools were considered that doctors had already used to de-identify their data for other purposes.

The consortium identified the CTP Anonymizer, an open-source, easy-to-install and user-friendly de-identification tool that could be used by the data providers. CTP Anonymizer offers the creation of a custom de-identification protocol through its various functionalities that can be executed on the DICOM fields, to de-identify them. The next step was to collaborate with both data providers, AI developers, and the legal partner of the project, to determine what the custom de-identification protocol would look like. The main pillar of this custom protocol, the INCISIVE de-identification protocol, was finding the balance between the usability of the data by the AI developers and keeping the data as private as possible. By combining the result of this collaboration and the information from other existing protocols, we implemented our own protocol.

The main focus of this protocol was to de-identify the name and identifier of the patient, as well as the unique IDs and dates that exist in various fields of the DICOM images. For the name and identifier of the patient, a naming convention was proposed, which followed the format: 0xx-xxxxxx.The first part (three-digit number), would be unique for each data provider and would be assigned to them prior to the de-identification process. The second part (a six-digit number after the '-') would be a number starting from 000001 and increasing sequentially for every new patient that was inserted in the CTP Anonymizer. This value would be the same for both the name and the unique identifier of the patient. For other different identifiers, a hash function would be applied using the de-identified patient ID as a seed. For the dates, it was mandatory to keep the original offset between consecutive examinations of the patient the same after the de-identification process.

A hash function was applied for the IDs, while a more elaborate technique was followed for the dates. It was crucial to modify the dates in a way that the original offset between different examinations of the patient remained the same after de-identification. For this purpose, the offset was computed using a hash function based on the patient id and then applied to the original dates. Other DICOM fields that might contain information leading to patient identification

and were not useful for the AI developers were either removed completely or replaced with a zero-length value. All of the transformations were provided through the CTP Anonymizer tool and were configured in a single de-identification script shared with the Data Providers.

The next step was to create own de-identification tool. This tool was based solely on the NEMA protocol and gives the Data Providers the option to select the level of privacy they want to apply to their data. This is achieved through various options inside the tool which the Data Provider can use to remove or de-identify different DICOM fields.

Challenges & Solutions

During data de-identification, several challenges were identified that needed to be tackled. The main problems included the definition and use of the right term for data de-identification and the implications of each (from both legal and technical sides), the homogeneity of the DICOM data, the complexity of the DICOM and NEMA protocols, and the non-unique identifiers for some patients.

Data De-identification terminology: A main takeaway was that the terms pseudonymization and anonymization, which in some cases are used interchangeably, have significant differences that affect both their implementation. For example, if the data is pseudonymized, it is feasible to add new data points for the same data object at a later time, while in anonymized data, such a thing is not feasible. After several discussions with our legal partner and taking into consideration the requirements during the term of the project, the data provided by most of the data providers to technical partners for the AI model training was considered to be pseudonymized (although the codes were never shared by the data contributor with other partners), rather than anonymized. Accordingly, we considered that the requirements of GDPR are applicable to handling such data during the project. Since after the project new data points will not be added, INCISIVE is developing an approach to anonymize the data for post project use.

Homogeneity of the DICOM data: Each data provider had different machines for performing examinations, and this led to DICOM images with different DICOM fields. As a result, the de-identification protocol had to be constructed in a way that could handle all the different DICOM fields, and in many cases, it had to be customized for each data provider separately.

Complexity of the DICOM protocol: As non-medical partners, the first difficulty we encountered when studying the DICOM protocol was that we could not easily interpret medical and technical terms related to the use of medical equipment. It became clear that the help of the data providers was mandatory in order to understand and clarify some of these definitions, and even then, the extent of the DICOM protocol did not allow us to fully understand it completely.

Non-unique identifiers: In some countries, patients performed their medical examinations in different facilities, which used different identifiers for the same patient in each facility. However, these examinations were collected by a single data provider, and thus we had to deal with multiple identifiers for a single patient. In these cases, an additional tool had to be used (DICOMEditor) to group these examinations and assign a single identifier for each patient in the DICOM fields.

ProCAncer-I

The core objective of the ProCancer-I project is the development of an imaging repository hosting a very large number (approximately 17.000) of anonymized prostate multi-parametric

(mp) MRI (T2-weighted, DWI, DCE images and resulting ADC parametric maps) or bi-parametric MRI examinations (T2-weighted, DWI and ADC) complying with qualitative and quantitative requirements, originating from 9 clinical centers geographically dispersed across Europe. Importantly, this vast volume of data, according to the patient's medical status, had to be divided according to the specific clinical use case (nine in total) that they are relevant to and provide the amount of additional clinical information that is required for solidly establishing the ground truth for each use case. As a consequence, an umbrella scenario that could be horizontally applied for registering the entire volume of patients was not appropriate for the needs of ProCancer-I. Each clinical endpoint required the DICOM images from an mp- or bi-parametric MRI of the prostate along with a carefully specified list of clinical or pathological information in order to formulate a valid entry for the Prostate NET repository. A sine qua non-condition for each contribution was the inability to trace back the patient's identity, even if other sources of information were made available.

De-identification process

To serve this purpose, a double-level anonymization approach was defined, comprising two discrete steps, the first step is completed locally by each data provider, whereas the second step, is horizontally applied to all shared data during the data upload in the data repository. Regarding the first step (blacklisting), the clinician is given the opportunity to apply their established anonymization workflows, while for the second step of the anonymization workflow (whitelisting), common rules were defined by the consortium and were horizontally applied to all data. The anonymization profile thus established focused on limiting the amount of information to those attributes that are explicitly relevant to the 9 use cases stated in the ProCancer-I proposal on the one hand, but also taking care not to compromise the data value for possible future uses on the other. The data ingestion workflow in ProCAncer-I is diagrammatically shown in Figure 4.

Identifier anonymization. The complete process of data preparation and uploading is initiated by preparing a folder to host the DICOM images which will then be liaised to the clinical and pathological information. The case's final identity is defined by the parameter PCa followed by a hash string generated from the site ID and the original patient ID and is attributed to each case during the second anonymization stage and kept thereafter.

Step 1. On-Premise. (Actor: Clinician). In this step, the necessary DICOM imaging series are extracted by authorized local users.

Step 2. Cloud Staging Area. (Actor: Clinician). In this staging area, imaging and clinical data are integrated and horizontally processed by an anonymization script (configured for each clinical partner separately) to guarantee that no unuseful, harmful, or possibly harmful information has escaped into the final repository.

Image anonymization. Since all imaging data comply with the DICOM standard, the image anonymization procedure was guided by the DICOM Committee Supplement 142-Clinical trial de-identification profile [**Error! Reference source not found.**] along with its updates [**Error! Reference source not found.**]. During the integration of the images with the clinical information, the list of tags is horizontally modified complying with the commonly agreed rules among the consortium. These rules were the end result of discussions held to reach a common understanding of the possible pitfalls concerning a very aggressive anonymization strategy also embracing the different legal and ethical engagements of each clinical partner. Moreover, the

issue of private tags was raised and the specific list of proprietary information included in the DICOM header was specified in order to retain the specific tags across the whole process.

More specifically, concerning the dates of examination an offset was defined from the original examination date, and where applicable, the relevant temporal distance between events (counted in a number of days) was calculated. Since the outcome of the project is related to vendor-specific and vendor-neutral models, it was important to keep the vendor information, while the institution name was not considered significant and presented a degree of risk to be combined with other tags and thus limit the number of individuals related to the specific information. Information concerning the acquisition parameters was kept (repetition time, echo time, etc.), as this information is strongly related to the data quality which is an important parameter for future data exploitation. In order to remove free text strings that may have been written in the native language giving away the site identity, this field was replaced with “mpMRI prostate” for every case. Moreover, for tag values that are characterized by extreme accuracy, such as time of acquisition exposing information to the detail of a second or even subdivisions of a second, care is taken to obscure it in order not to drastically restrict the number of individuals, once relevant information is provided by the clinical site.

The method chosen to apply this common approach was the command line tool or RSNA CTP Anonymizer by a set of rules in the designated script file.

Clinical data anonymization. An eCRF form serves to integrate a predefined number of optional and mandatory fields relevant to each use case and is either manually provided by the actor for single patients or derived from a text file for batch, before being forwarded to the cloud staging area through an encrypted communication channel. However, no automatic extraction of information is required, minimizing the risk of any PHI escaping into the public area. The clinical information is linked and recognized after the same identifiers as the imaging data. The dates of each clinical event are relatively counted from a specific critical diagnostic point and are not given in absolute dates. Moreover, care is taken not to receive information from the clinical aspect that has been restricted or modified during imaging data processing, such as exact birth date or weight. The information is stored under the OMOP common data model and its extensions [**Error! Reference source not found.**, **Error! Reference source not found.**].

Challenges & solutions

Challenges addressed during the anonymization task can be summarized in:

Heterogeneity among headers from different vendors/software releases: As herein described, the scope of the second anonymization layer was to keep a common list of attributes that are present in all clinical sites and exclude information that does not consistently appear in all DICOM headers

Burned-in information in secondary captures: The selected tool has the ability to detect and erase burnt-in information. However, a number of known image or series attributes were examined in order to raise the user's awareness for images suspicious of being secondary captures conveying burned-in information, i.e., single images.

Handling of Private DICOM tags: Private DICOM tags contain information proprietary to the vendor and therefore may contain information that is related to the patient or site identity. However, some important acquisition information may reside in private tags. Such information

was identified and only a very restricted number of private tags selected for their value in future data use were retained in the final DICOM tag list.

PRIMAGE

The PRIMAGE project aims to build a centralized cloud-based platform with data coming from different sources across Europe with the objective of building a clinical decision support system (CDSS) to help in the diagnosis, follow-up, and treatment planning of patients with neuroblastoma and diffuse intrinsic pontine glioma (DIPG). The PRIMAGE database was built by an internal database with data coming from consortium partners and an external database which was built with the help of external collaborators that were engaged during the execution of the project. Once collected, the internal database was used to train and internally validate AI-based tools developed to predict different clinical endpoints (e.g., Overall Survival, Event Free Survival, among others), while the external database was used for the external validation of the AI models in a completely independent database. Therefore, using the PRIMAGE platform, based on Quibim Precision, data providers uploaded all their cases by providing both imaging data (MRI, CT, PET, and MIBG scans) and clinical data which was shared in a structured way through an eCRF.

De-identification process

The overall de-identification process of PRIMAGE is shown in Figure 5.

Patient identifier. The PRIMAGE de-identification process was based on either anonymization or pseudonymization, depending on the data provider's preferences.

Anonymization: During the data ingestion process, the PRIMAGE platform generated a new code, following the structure *XX_ProjectName_YYYY*, where *XX* was the site code given to the specific data provider in the PRIMAGE platform, *ProjectName* was either Neuroblastoma or DIPG, and *YYYY* was a unique number given to the specific case.

Pseudonymization: The EUPID (European Unified Patient Identity) was selected as the pseudonymization tool in the PRIMAGE platform. With EUPID, given some patient information, such as the patient's name, surname, and date of birth or the pseudonym from a different context (e.g., SIOPEN-r-net clinical trial), a phonetic hash was created and used to create the patient's pseudonym. Therefore, the pseudonyms used allow the data linkage across different projects and avoid duplications (the pseudonym is the same as long as the same patient data is introduced), however, it’s related to hashed patient data and not real data. Figure 5 shows the pseudonym generation process, where, (1) the user introduces the patient information in the PRIMAGE platform web interface, (2) with this information a phonetic hash is created and (3) sent to EUPID services, and finally, (4) the new patient pseudonym is sent back to the PRIMAGE platform and (5) shown to the user together with some messages telling the user if (1) there is no matching and a new pseudonym has been created; (2) there is a full match with a previously created pseudonym and, therefore, the same ID is used; (3) there is a partial match, so the user needs to decide to link with the other pseudonym or to create a new one; or (4) there are conflicts with multiple pseudonyms.

Image de-identification. When uploading an imaging study, the previously created identifier is used to substitute personal data in the DICOM files such as the Patient Name or the Patient ID, and all the DICOM tags with sensitive information as stated in the DICOM standards PS3.15 are

removed or emptied from the uploaded files. Additionally, the PRIMAGE platform includes a tool to remove any sensitive burned data within the image. By drawing a rectangle on a specific region, the tool erases this area of the image before uploading by assigning background pixel values to the whole delineated region.

Clinical data de-identification. Clinical data is ingested either manually through an eCRF or automatically through the API of the PRIMAGE platform, therefore the clinical data is also associated with the corresponding patient identifier. There are some fields, such as the birthdate, that are considered sensitive information. Therefore, patient age at diagnosis is automatically calculated when both the diagnosis date and birthdate are introduced, being those two values not stored in the database. All the data is introduced in a structured manner avoiding, as much as possible, free text fields to avoid any PHI being introduced by mistake.

Challenges and Solutions

Differences in de-identification process needs and requirements. Data from the SIOPEN-r-net database was already pseudonymized using EUPID (European Unified Patient Identity). EUPID allows the registration and pseudonymization of patients as well as the linkage of the different datasets without the need to use directly identifying data elements. In these clinical trials, pseudonymization was a prerequisite for merging clinical variables with associated image data (MRI, CT, PET, mIBG Scans) and further tumor-biological data, while respecting the data protection requirements. However, some other centers preferred a complete anonymization of the cases within the PRIMAGE platform. Therefore, to overcome this challenge, both solutions were implemented.

Already anonymized imaging studies. Some clinical centers, before uploading the imaging studies to the PRIMAGE platform, applied their own anonymization processes. There were some cases where DICOM tags such as the study description or the series description were also anonymized making the identification of the specific series (i.e., T1w, T2w, DWI) difficult. To overcome this limitation, an AI-based classifier was developed which, given some DICOM metadata such as the sequence variant, MR acquisition type, echo time, and repetition time, among others, the specific MR sequence was given.

Burnt information in the DICOM images. As pointed out in other projects, some DICOM images include sensitive information within the pixels of the image. To avoid uploading this information to the central repository the following steps could be found during the uploading process: 1) When a DICOM study is selected for its upload, the user has a preview where all the series to be uploaded are listed. The user can previsualize each image and discard any from the upload. 2) If the image wants to be uploaded, a manual anonymization step was incorporated. Therefore, the user can delineate the area where sensitive information appears in the image being this area erased before uploading.

Appendix 2

Table S1. DICOM Tags modified by ProCAncer-I, CHAIMELEON, PRIMAGE, INCISIVE and EuCanImage.

DICOM Tags modified			
InstitutionName	(0008,0080)	StudyID	(0020,0010)
InstitutionAddress	(0008,0081)	RequestingPhysician	(0032,1032)
InstitutionCodeSeq	(0008,0082)	SpecialNeeds	(0038,0050)
ReferringPhysicianName	(0008,0090)	ScheduledPerformingPhysicianName	(0040,0006)
ReferringPhysicianAddress	(0008,0092)	PreMedication	(0040,0012)
ReferringPhysicianPhoneNumbers	(0008,0094)	NamesOfIntendedRecipientsOfResults	(0040,1010)
ReferringPhysicianIdentificationSeq	(0008,0096)	IntendedRecipientsOfResultsIdentificationSeq	(0040,1011)
PhysicianOfRecord	(0008,1048)	PersonIdentificationCodeSeq	(0040,1101)
PhysicianOfRecordIdentificationSeq	(0008,1049)	PersonAddress	(0040,1102)
PerformingPhysicianName	(0008,1050)	PersonTelephoneNumbers	(0040,1103)
PerformingPhysicianIdentificationSeq	(0008,1052)	OrderEnteredBy	(0040,2008)
NameOfPhysicianReadingStudy	(0008,1060)	OrderEntererLocation	(0040,2009)
PhysicianReadingStudyIdentificationSeq	(0008,1062)	HumanPerformerName	(0040,4037)
OperatorName	(0008,1070)	VerifyingObserverName	(0040,A075)
OperatorIdentificationSeq	(0008,1072)	PersonName	(0040,A123)
PatientName	(0010,0010)	PresentationCreatorName	(0070,0084)
PatientID	(0010,0020)	ReviewerName	(300E,0008)
IssuerOfPatientID	(0010,0021)	InterpretationRecorder	(4008,0102)
PatientBirthDate	(0010,0030)	InterpretationTranscriber	(4008,010A)
OtherPatientNames	(0010,1001)	InterpretationAuthor	(4008,010C)
OtherPatientIDSeq	(0010,1002)	PhysicianApprovingInterpretation	(4008,0114)
PatientBirthName	(0010,1005)	DistributionName	(4008,0119)
PatientAddress	(0010,1040)		
PatientMotherBirthName	(0010,1060)		
ResponsiblePerson	(0010,2297)		
